# Enhancing effect of oregano essential oil and *Bacillus subtilis* on broiler immune function, intestinal morphology and growth performance

**DOI:** 10.1186/s12917-024-03960-w

**Published:** 2024-03-22

**Authors:** Yehia El-Sayed, Waleed Khalil, Nada Fayez, Abdel-Fattah Mohamed Abdel-Fattah

**Affiliations:** Pharmacology Department, Faculty of Veterinary Medicine, Suez Canal University, Ismailia, 41522 Egypt

**Keywords:** Probiotic, Phytobiotic, Growth performance, Broiler, Immunity

## Abstract

The present study evaluated the effect of two categories of feed additives on chicken performance through immunological and intestinal histo-morphometric measurements. A total of 150 one-day-old male broiler chicks (Cobb) were randomly assigned to three groups. Group I received a non-supplemented basal diet. While groups II and III were treated with a basal diet supplemented with oregano essential oil (OEO) and *Bacillus subtilis*, respectively, in water for 28 days. Blood samples were taken at 6, 18 and 28 days for hematological analysis, phagocytosis, lymphocyte proliferation and measuring antibody responses. Additionally, growth performance indices were recorded weekly. The results showed that groups supplemented with OEO and *B. subtilis* improved growth performance expressed by a significant increase in weight gain (*P* < 0.05), with a significant reduction (*P* < 0.05) in feed conversion ratio (FCR). Hematological findings indicated a significant increase in blood parameters as well as a significant increase in phagocytic % & phagocytic index at all time points with a greater probiotic effect. On the other hand, OEO produced a significant increase in lymphocyte proliferation at 18 & 28 days. Humoral immunity revealed a significant increase in serum antibody titer phytobiotic & probiotic-fed groups at time points of 18 & 28 days with a superior phytobiotic effect. The histological examination showed a significant increase in villi length, villi width, crypt depth & V/C ratio. In conclusion, these results indicated positive effects of *B*. *subtilis* & OEO on both growth and immunity and could be considered effective alternatives to the antibiotic.

## Introduction

In poultry production, protecting the gastrointestinal tract (GIT) and enhancing the birds’ health are the top priorities. The GIT serves as the first line of defense, providing a large surface area for food degradation and absorption; However, the GIT is vulnerable to pathogens and toxins that can penetrate the body by damaging the mucus layer [1]. Therefore, maintaining the GIT is crucial due to the potential harm caused by common toxins such as aflatoxin and lipopolysaccharide (LPS), which can damage intestinal cells, cause inflammation, trigger immune responses, and impair production. Thus, protecting the GIT from pathogen and toxin invasion and enhancing overall bird immunity are essential [2, 3].

The overuse of antibiotics has raised concerns due to compelling evidence that it has accelerated the emergence of antibiotic-resistant bacteria, which can transfer to humans and render antibiotics ineffective for both humans and animals [4]. The link between antibiotic resistance and medication use in food animals is still debated in the United States, but in Europe, concerns about the potential harm to human health have led to legislative action that has been in place for over 28 years. Despite ongoing debates, the European Union banned growth-promoting antibiotics for food animals in 2006 [5]. Many other governments have also restricted antibiotic use, leading to increased research into alternative feed additives for antibiotics. Potential alternatives include feeding prebiotic compounds, probiotic organisms, enzymes, herbs, essential oils, and acidifying feed using organic acids [6].

Phytogenics and phytochemicals are among the most promising feed additives due to their positive effects on regulating the immune response in chickens and pigs. As natural-sourced materials, phytochemicals are potential feed additives with multiple functions, including anti-inflammatory, anti-fungal, anti-viral, and antioxidative properties.

Probiotics are live microorganisms added to animal feed to establish a beneficial gut microflora [7]. Dietary supplementation with probiotics has been shown to enhance daily gain and feed intake, increase lactobacilli numbers, and reduce *E. coli* numbers [8]. Numerous studies have demonstrated that adding probiotics to broiler and turkey diets improves performance [9–11].

Phytogenic compounds have a complex mode of action, which has been a significant area of interest for those using these substances as feed additives. Therefore, recent analysis and development efforts have focused on understanding the role of phytogenic feed additives in enhancing animal performance and health [12–14]. The benefits of supplementing poultry feed with a combination of herbal essential oils and plant extracts are well-documented [15, 16]. Including phytogenic feed additives helps improve host immunity through interactions with the gut-associated lymphatic system [17, 18].

This study aimed to investigate the effects of some antibiotic alternatives on the growth performance, immunity, and histopathology of broiler chickens.

## Materials and methods

### Chicks and experimental design

A total of 150 male one-day-old Cobb broiler chicks were randomly distributed into three groups each with 5 replicates of 10 birds obtained from Al- Shrouk Poultry Company, Egypt. The birds were housed on the deep litter floor system (with hay from the day of the hatch) at a private farm. All housing areas, feeders, drinkers and heaters were cleaned and disinfected before the study. The environmental temperature was adjusted according to age. It was set at 33˚C for the first three days of age and then, decreased by 1˚C every three days until it reached 26˚C at the 4th week of age. Relative humidity was set at 60–70% throughout the study. Ventilation was controlled to maintain the birds’ comfort during the rearing period. Birds were provided 24 h of lighting on the first day then decreased by one hour daily until reaching four hours of darkness by the end of the experiment and checked three times daily (at 8 am, 2 pm and 10 pm) for food, water and mortality. Animal handling and care were conducted according to the National Research guidelines [19]. Birds received the basal diet (**CON**), with the phytobiotic product **(PHYTO)** or the probiotic product **(PRO).** The Probiotic product (**CLOSTAT® HC SP Dry** Kemin Industries Inc, USA**)** composed of *Bacillus subtilis PB6* 2 × 10^9^ units per gram oral powder was added in drinking water for 8 h daily. The Phytobiotic product **(Ropadiar® Solution**, Ropapharm International B.V, the Netherlands) consisted of oregano oil 20% solution and was included in drinking water (1 ml /5 liters) for 8 h daily. Rations were formulated as starter and grower diets. The chicks were fed formulated broiler starter basal rations from one day old to two weeks of age and then fed a grower ration until the end of the experiment at 28 days of age. The diet was formulated to meet the nutritional requirements recommended by the NRC [20] as shown in Table 1.

**Table 1:** Feed ingredients and calculated chemical analyses of basal diets

Item	Starter (0-14 day)	Grower (15-30 day)
Ingredients, %		
yellow corn (grains)	54	59.2
Soya bean meal (44%)	32.85	28
Corn gluten (62%)	6.5	6
Soybean oil	2.7	2.5
Dicalcium phosphate	1.46	1.52
Limestone	1.51	1.8
Premix	0.3	0.3
Common salt	0.3	0.3
(NaCl) DL Methionine	0.28	0.28
L-Lysine HCL	0.1	0.1
**Total**	**100**	**100**
**Chemical analysis (Calculated)**:		
ME, Kcal/kg	3200	3194
Crude protein (C.P %)	23	21
Calcium%	1.1	0.93
Available Phosphorus%	0.42	0.42
Lysine%	1.19	1.07
Methionine and Cysteine%	1.06	1.01

Each 3 Kg of premix contains vitamins: A: 12,000,000 IU; D3 2,000,000 IU; E: 10,000 mg; K3: 2000 mg; B1:1000 mg; B2: 5000 mg; B6:1500 mg; B12: 10 mg;

Biotin: 50 mg; Choline chloride: 250,000 mg; Pantothenic acid: 10,000 mg;

Nicotinic acid: 30,000 mg; Folic acid: 1000 mg; Minerals: Mn: 60,000 mg; Zn: 50,000 mg; Fe: 30,000 mg; Cu: 10,000 mg; I: 1000 mg; Se: 100 mg and Co: 100 mg.

### Growth performance parameters

Individual body weight and feed consumption per cage were recorded on 0, 7, 14, 21 & 28 days to calculate the daily body weight gain (DWG) [Final BW − initial BW], average daily feed intake (ADFI), feed conversion ratio (FCR) [ADFI to DWG], Total feed efficiency (FE) [DWG to ADFI], and these parameters were corrected for mortality.

### Blood sampling

At the 6th, 18th and 28th days of age, 5 birds in each group were randomly chosen for blood sampling. Three blood samples were collected directly into triplicate sterilized tubes, one containing heparin, another tube containing K_3_EDTA for the determination of hematological parameter values, and the third without anticoagulant. The sera were collected in Eppendorf tubes and stored at -20˚C to be used in the hemagglutination inhibition assay.

### Hematological study

Collected blood samples with K3EDTA anticoagulant from each group were used to perform differential leukocytic count (DLC) carried out as reported by Schalm et al. [21] and Gross and Siegel [22].

### Immunological studies

#### Hemagglutination inhibition test

Chickens were vaccinated ocular route at 8th day old against Newcastle (ND) and Avian influenza (AI) using NOBILIS® CLONE 30 vaccine (Intervet International B.V.35 Wim de örverstraat oxmeer, The Netherlands). Humoral immune response was investigated by detecting serum antibody titers against ND and AI viruses by hemagglutination inhibition test as described by Alexander and Chettle [23].

#### Phagocytic activity % and phagocytic index

Collected heparinized blood samples and Candida albicans were used to perform the phagocytic assay according to the method described by Jensch-Junior et al. [24]. Smears were prepared from viable leukocyte suspension incubated with inactivated *C. albicans*, followed by the evaluation of phagocytic activity & phagocytic index. The total number of phagocytes which ingested candida was determined to calculate the percentage of phagocytosis and phagocytic index.

#### Lymphocyte proliferation activity

The proliferation activity was determined by measuring mitochondrial activity using the MTT [3(4,5-dimethylthiazal-2-yl)-2,5-diphenyl tetrazolium bromide] reduction method according to Rai-el-Balhaa et al. [25]. After the collection of lymphocytes, they were cultured in a 96-well plate, incubated for 4 h in the presence of MTT (5 mg/ml) (Sigma-Aldrich, St. Louis, MO, USA) followed by the addition of 0.1 ml dimethyl sulfoxide (DMSO) (Merck, Germany). Then the crystals were dissolved and adding 10% SDS in 0.01 M HCL was during an additional incubation period of 16 h. The absorbency was read at 590 nm in an enzyme immunoassay multi-well photometer or ELISA reader.

The increase in absorbency, after subtracting the background absorbency, was referred to as “MTT units”.

#### Histomorphometry measurements of the intestinal tract (jejunum)

At 6,18 and 28 days of the experiment, The birds were euthanized using cervical dislocation following the mechanical cervical dislocation method [26]. Subsequently, the abdomen of each carcass was opened, and five-cm long segments were extracted from the jejunum, these segments were then rinsed with normal saline and immersed in formalin. After tying them with threads from both sides, they were placed in 10% buffered formalin for fixation to calculate crypt depth and villus height to crypt depth ratio [27]. Morphometric analyses of the intestinal epithelium were conducted using TS view software and ImageJ software with a standard calibrated stage micrometer and a calibrated standard digital microscope camera (Tucsen digital camera) mounted on an Olympus CX21 microscope, featuring a resolution of 5 MP (2592 × 1944 pixels per image). All slides were captured at a Power Field of 100x, utilizing the UIS optical system (Universal Infinity System). WS [28] was employed to determine villus height (VH, µm), measured from the top of the villus to the top of the lamina propria. Crypt depth (CD, µm) was defined as the depth of the invagination between the adjacent villi [29].

#### Statistical analysis

The obtained data were statistically analyzed by one-way analysis of variance (ANOVA) considering *P* < 0.05 using SPSS 14.0.0 software [30]. The significant differences were subjected to Duncan multiple range tests to compare the means. All data are expressed as arithmetic means ± standard errors except for the HI where the harmonic mean ± standard error is used.

## Results

### Growth performance parameters

#### Live body weight (g), weight gain (g), feed intake (g) and feed conversion ratio

The data showed that the mean values of live body weight for both probiotics and phytobiotic-fed groups were significantly higher (*p* < 0.05) than those of the control group during the entire experiment period. Similarly, until day 21, both treated groups showed a significant increase in body weight gain that was higher than the control group. Regarding feed intake, and FCR, the findings demonstrated that, at the end of the 1st, 2nd and 3rd weeks of age, the control group had a significant (*P* < 0.05) reduction in feed intake compared to both treated groups. However, by the end of the 3rd week, the feed intake was significantly reduced in the probiotics and phytobiotic-treated groups compared to the control group. Also, FCR decreased significantly at the end of the 2nd week, Table 2.

**Table 2:** Effects of probiotic & phytobiotic on the growth performance parameters of broilers

Age/Day	Group			
	Growth Parameters	(CON) Control	(PHYTO) Phytobiotic	(PRO) Probiotic
0 day	• Initial body weight	41.35±0.21^a^	41.35±0.38^a^	41.41±0.12^a^
0-7 day	• body weight gain	127.48±1.45^c^	142.15±1.22^b^	142.92±0.80^a^
	• cumulative feed intake	166.67±1.33^b^	190.33±2.95^a^	194.05±1.09^a^
	• cumulative FCR	0.99±0.01^b^	1.04±0.02^a^	1.05±0.01^a^
7-14 day	• body weight gain	226.17±1.44^a^	231.17±1.60^b^	259±1.65^b^
	• cumulative feed intake	404.63±2.28^b^	454.98±4.71^a^	455.82±1.19^a^
	• cumulative FCR	1.02±0.01^b^	1.1±0.01^a^	1.03±0.01^b^
14-21 day	• body weight gain	360.33±1.37^c^	409.50±1.91^b^	440.0±2.08^a^
	• cumulative feed intake	1041.52±6.89^a^	1090.28±6.80^b^	1096.77±9.04^c^
	• cumulative FCR	1.38±0.01^a^	1.32±0.01^b^	1.24±0.01^c^
21-28 day	• body weight gain	501.63±1.54^c^	555.5±2.79^b^	610.65±1.72^a^
	• cumulative feed intake	2023.90±11.14^a^	1936.63±8.39^b^	1942.83±9.79^c^
	• cumulative FCR	1.61±0.01^a^	1.4±0.01^b^	1.3±0.01^c^

FCR = feed conversion ratio.

Values are expressed as means ± standard error (SE); *n*=5.

Means within the same row with different superscripts are significantly different (*P*<0.05).

### Hematological studies

The results indicated that there were significant increases (*P* < 0.05) in the WBC counts and lymphocyte percentages in experimental broiler chicks as a result of dietary supplementation with probiotics and phytobiotics compared to the control groups. Besides, the phytobiotic-supplemented group had the highest WBC count and lymphocyte count than other groups.

**Table 3:** The effect of probiotic & phytobiotic on differential leukocytic count of the experimental chickens

Time point (days)	Parameter						
	Group	WBCs(X10^3/cmm)	Lymphocytes	Heterophils	Monocytes	Eosinophils	Basophils
6^th^ day	(CON)	18.75±0.36^b^	10.23±0.29^b^	6.46±0.11^a^	1.44±0.05^b^	0.50±0.02^a^	0.31±0.02^a^
	(PHYTO)	19.30±0.31^ab^	10.50±0.33^ab^	6.48±0.16^a^	1.49±0.06^ab^	0.47±0.05^a^	0.35±0.04^a^
	(PRO)	20.29±0.35^a^	11.19±0.20ab	6.63±0.25a	1.58±0.02a	0.54±0.06a	0.35±0.04a
18^th^ day	(CON)	19.25±0.37^b^	10.49±0.34^b^	6.50±0.22^a^	1.44±0.05^b^	0.46±0.04^a^	0.36±0.03^a^
	(PHYTO)	20.59±0.35^a^	11.10±0.19b	6.94±0.25a	1.60±0.07ab	0.54±0.03a	0.41±0.04a
	(PRO)	21.58±0.35^a^	11.96±0.23a	7.01±0.14^a^	1.63±0.04^a^	0.52±0.04^a^	0.43±0.04^a^
28^th^ day	(CON)	19.09±0.035^c^	10.25±0.33^c^	6.45±0.16^c^	1.51±0.05^b^	0.51±0.02^a^	0.38±0.02^a^
	(PHYTO)	21.04±0.46^b^	11.10±0.19b	7.01±0.10b	1.68±0.06a	0.52±0.04a	0.41±0.02a
	(PRO)	22.74±0.14^a^	11.96±0.23^a^	7.48±0.7^a^	1.81±0.04^a^	0.56±0.02^a^	0.42±0.02^a^

Values are expressed as means ± standard error (SE); *n*=5.

Means within the same row with different superscripts are significantly different (*P*<0.05).

WBCs= White Blood Cells.

**Table 4:** The effect of probiotic & phytobiotic on some hematological parameters of the experimental chickens at 28^th^ day

Group	Parameter						
	RBCs (X10^6/cmm)	Hb g/dl	PCV %	Platelets (X10^3/cmm)	MCV (fl.)	MCH (pg)	MCHC (g/dl)
(CON) control	2.85±0.13^b^	7.90±0.18^c^	28.56±0.72^c^	29.20±1.07^c^	100.69±2.20^a^	27.86±0.68^a^	27.67±0.15^a^
(PHYTO) phytobiotic	3.62±0.18^a^	9.788±0.28^b^	35.38±0.73^b^	34.4±1.29^b^	98.416±3.43^a^	27.18±0.74^a^	27.648±0.22^a^
(PRO) probiotic	3.99±0.13^a^	10.87±0.30^a^	38.36±0.46^a^	41.00±1.14^a^	96.48±2.27^a^	27.27±0.22^a^	28.31±0.53^a^

Values are expressed as means ± standard error (SE); *n*=5.

Means within the same row with different superscripts are significantly different (*P*<0.05).

RBCs= Red Blood Cells; Hb= Hemoglobin; PCV= Packed Cell; MCV= Mean Corpuscular Volume: MCH= Mean Corpuscular Hemoglobin; MCHC= Mean Corpuscular Hemoglobin concentration.

Similarly, the results of the blood picture on day 28 of the experiment showed that the phytobiotic-supplemented group had a remarkable increase (*P* < 0.05) in RBC count, Hb, PCV% and platelet count, followed by the probiotic group (*P* < 0.05) then the control group, Tables 3 and 4.

### Broiler immunological studies

#### Humoral immunity by detecting the antibody titers against NDV. By hemagglutination inhibition (HI) assay

The phytobiotic-fed group showed the highest increase (*P* < 0.05) in the HI antibody titers against NDV. followed by the probiotic group then the control, Table 5.

**Table 5:** Effects of probiotic & phytobiotic on humoral immune response estimated by detecting serum antibody titers against NDv.by hemagglutination inhibition test

Age/Day	Group		
	(CON) Control	(PHYTO) Phytobiotic	(PRO) Probiotic
6^th^ day	2.31±0.19^a^	1.94±0.40^a^	1.71±0.47^a^
18^th^ day	2.61±0.30^b^	4.35±0.40^a^	3.26±0.58^ab^
28^th^ day	1.25±0.19^a^	1.94±0.40^a^	1.54±0.43^a^

Values are expressed as means ± standard error (SE); *n*=5.

Means within the same row with different superscripts are significantly different (*P*<0.05).

ND = Newcastle disease virus.

#### Phagocytic %, index and lymphocyte proliferation activity

At 6,18 and 28 days of the experiment, the phytobiotic-supplemented group showed a significant increase (*P* < 0.05) in both Phagocytic % and index followed by the phytobiotic group than the control. On the other hand, OEO produced a significant increase in lymphocyte proliferation at time points of 18 & 28 days, Table 6.

**Table 6:** Effects of probiotic & phytobiotic on phagocytic %, index and lymphocyte proliferation activity

Group	Parameter			
	Age/days	Phagocytic %	Phagocytic index	lymphocyte proliferation activity
(CON) Control	6^th^ day	57.8±1.5^c^	2.72±0.07^b^	3.15±0.11^a^
	18^th^ day	58.6±1.94^b^	2.68±0.10^c^	3.48±0.13^b^
	28^th^ day	68.0±1.41^a^	3.56±0.16^a^	2.13±0.33^b^
(PHYTO) Phytobiotic	6^th^ day	69.8±1.56^c^	3.46±0.12^b^	2.51±0.08^b^
	18^th^ day	70.40±1.63^b^	4.10±0.34^a^	4.18±0.17^a^
	28^th^ day	73.0±1.58^a^	4.34±0.16^a^	3.77±0.52^a^
(PRO) Probiotic	6^th^ day	81.80±1.28^b^	4.14±0.09^b^	3.40±0.13^a^
	18^th^ day	80.40±1.54^c^	4.46±0.23^a^	3.98±0.12^a^
	28^th^ day	82.40±1.63^a^	4.78±0.12^a^	2.00±0.28^b^

Values are expressed as means ± standard error (SE); *n*=5.

Means within the same row with different superscripts are significantly different (*P*<0.05).

### Intestinal histomorphometric measurements

**Fig. 1:**
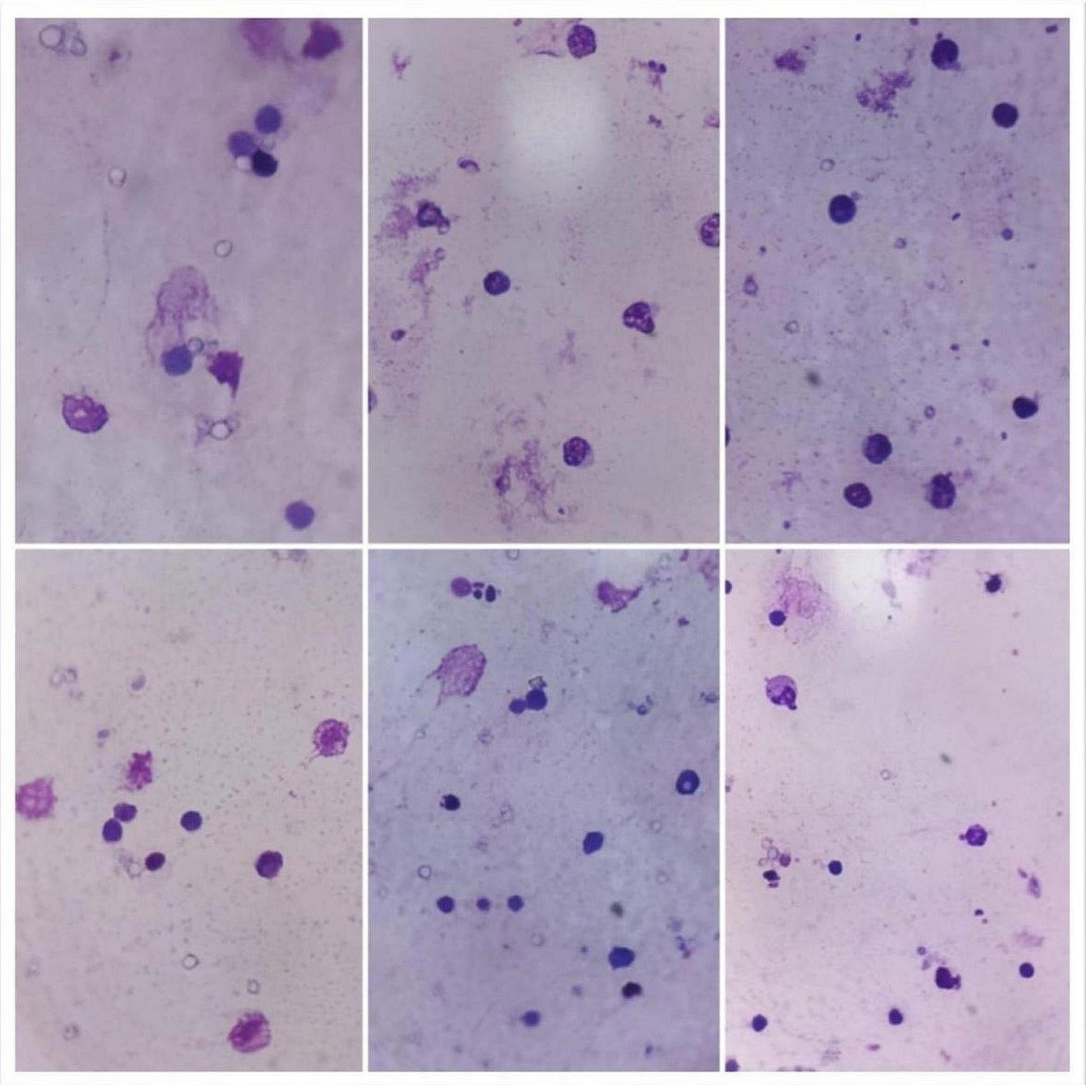
phagocytes assessment in control group at different ages

**Fig. 2:**
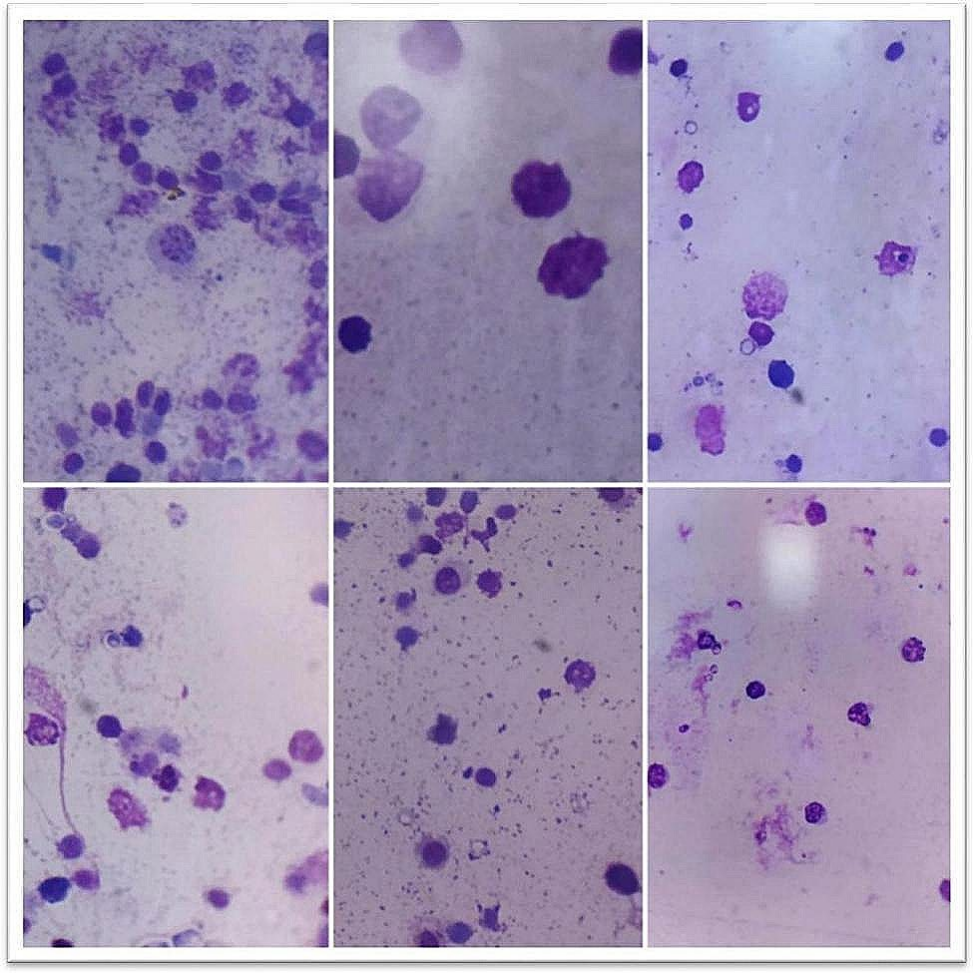
phagocytes assessment in phytobiotic group at different ages

**Fig. 3:**
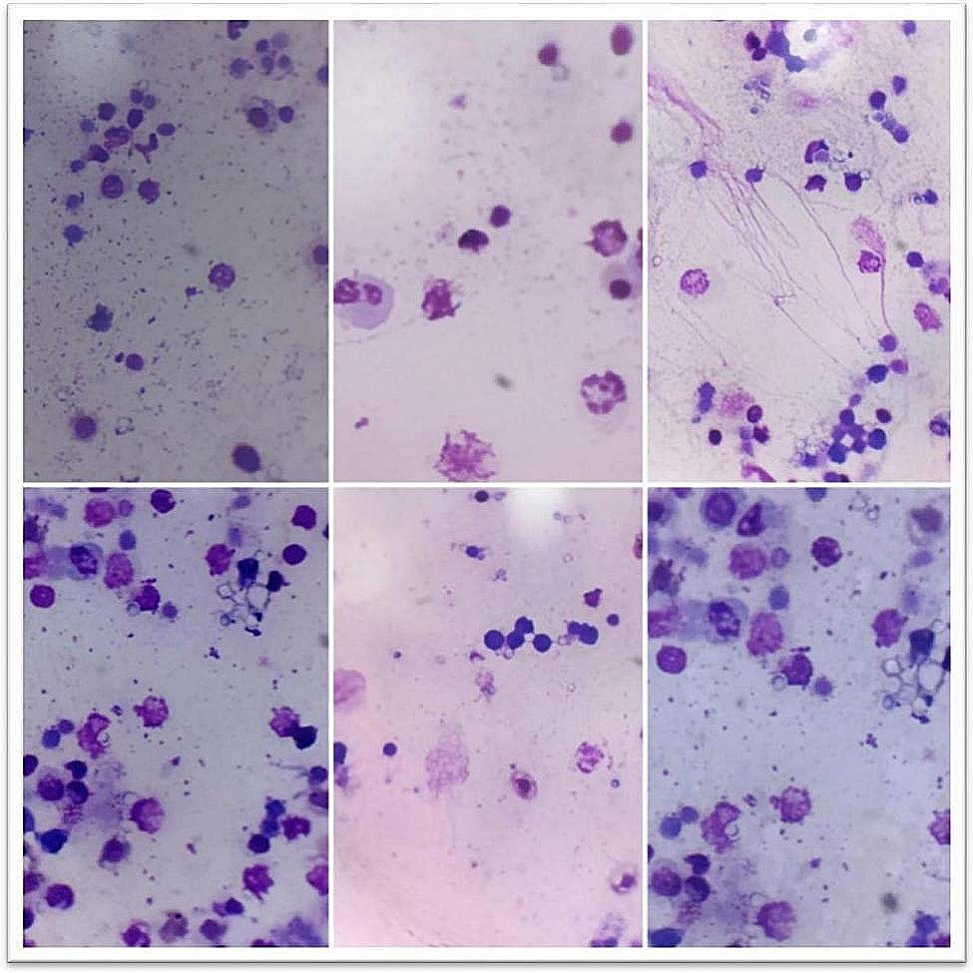
phagocytes assessment in probiotic group at different ages

**Fig. 4:**
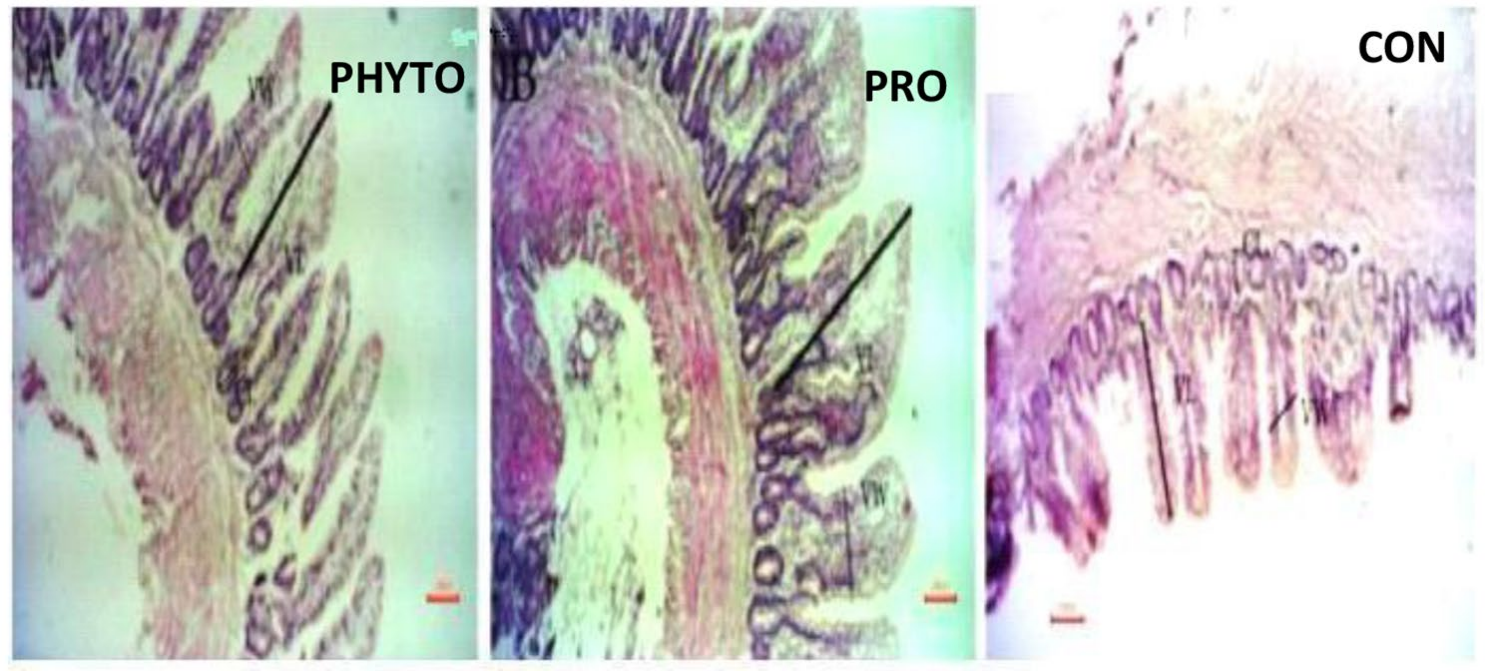
effect of phytobiotic and probiotic feed additives on jujinal microstructure of

**Fig. 5:**
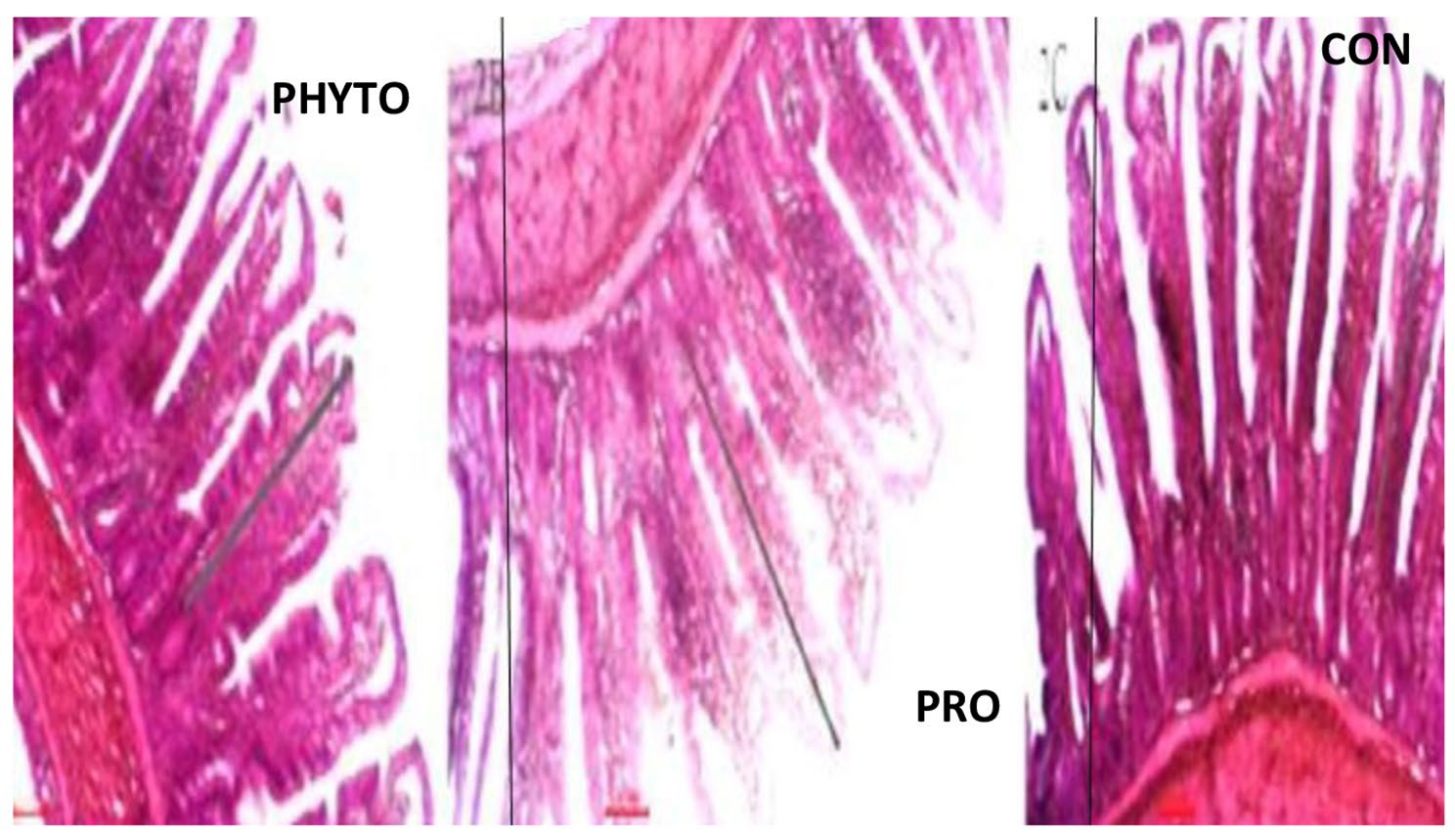
effect of phytobiotic and probiotic feed additives on jujinal microstructure of broiler chicken at 18th day of age

**Fig. 6:**
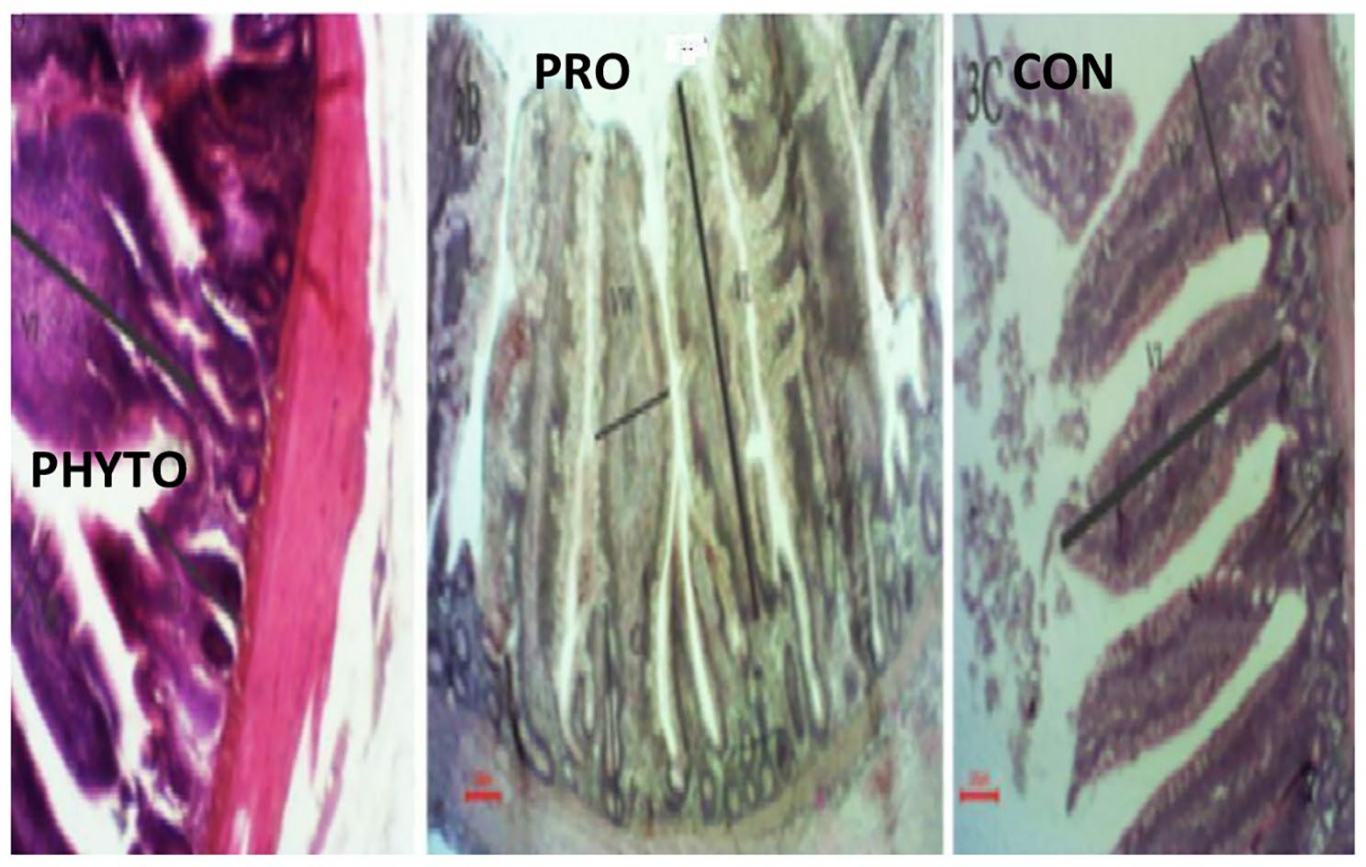
effect of phytobiotic and probiotic feed additives on jujinal microstructure of broiler chicken at 28th day of age

Regarding the intestinal villi parameters, the probiotic-supplemented group exhibited the highest significant (*P* < 0.05) levels. Additionally, the groups supplemented with phytobiotics showed a significant increase (*P* < 0.05) in jejunal villous height than the control group, Table 7 and Figs. 1, 2, 3, 4, 5, and 6.

**Table 7:** The effect of probiotic & phytobiotic on jejunal histology of the experimental chickens

Time point (days)	Parameter				
	Groups	VL (Micron)	VW (Micron)	Crypt depth(Micron)	v/c ration
6^th^ day	(CON)	282.24±8.04^c^	121.50±2.96^b^	99.30±3.16^c^	2.91±0.14^b^
	(PHYTO)	329.53±10.16^b^	136.40±5.02^b^	114.83±4.66^b^	2.95±0.17^b^
	(PRO)	537.91±23.81^a^	207.71±7.82^a^	156.94±2.77^a^	3.46±0.19^a^
18^th^ day	(CON)	434.95±13.77^c^	140.00±10.40^c^	165.79±8.13^b^	3.07±0.18^a^
	(PHYTO)	492.37±11.65b	212.69±7.08b	133.05±5.51c	3.34±0.16a
	(PRO)	627.52±22.17^a^	252.39±5.57^a^	224.47±8.91^a^	3.31±0.17^a^
28^th^ day	(CON)	800.93±32.68^b^	181.04±6.47^c^	154.29±6.92^b^	5.34±0.35^a^
	(PHYTO)	903.39±66.34b	225.78±7.74b	148.19±8.06b	6.43±0.68a
	(PRO)	1423.10±86.11^a^	309.66±4.87^a^	285.97±13.14^a^	5.07±0.40^a^

Values are expressed as means ± standard error (SE); *n*=5.

Means within the same row with different superscripts are significantly different (*P*<0.05).

VL= villous length; VW= villous width; V/C= villous length: crypt depth ratio.

## Discussion

Due to various issues such as the widespread presence of drug-resistant bacteria, antibiotic residues in animal tissue and environmental concerns, arising from the overuse of antimicrobials in food animals & poultry, an alternative strategy to disease management involves utilizing growth promotors and immunostimulants derived from natural sources instead of antibiotics.

The study demonstrated that phytobiotics, such as OEO, and probiotics, like B. subtilis, exhibited a significant increase (*P* < 0.05) in final body weight, total body weight gain, and a notable reduction in both feed intake and FCR compared to the control group by the end of the experiment. These beneficial effects of probiotics and phytobiotics could be attributed to their ability to create a favorable environment in the intestine, influencing the intestinal microbiota by promoting beneficial bacteria over pathogenic ones and enhancing intestinal microstructure and health. These observations are supported by the data published by several authors [31–36] for both phytobiotic and probiotic.

Probiotics were found to yield the best results in terms of body performance compared to phytobiotics and control groups, which aligns with previous findings [37]. The mechanisms through which phytobiotics, OEO, and probiotics (B. subtilis) work are complex and not fully defined. Proposed mechanisms include the production of antimicrobial compounds, competitive adhesion to mucosa and epithelium, reinforcement of the gut epithelial barrier, and modulation of the immune system [38, 39]. Several studies demonstrated that Probiotics alter the gut environment and improve the function of the gut barrier, stimulate the immune system, fortify the good intestinal flora, and inhibit infections through competitive exclusion, which in turn promotes growth. Following the addition of probiotics, the non-pathogenic bacteria from the probiotics compete with the pathogenic bacteria in the gut for nutrients, colonize the intestine, prevent the growth or establishment of harmful bacteria, and secrete digestive enzymes (such as galactosidase, amylase, etc.), which aid in increased nutrient absorption and enhance animal growth performance [38, 40]. Even in Cob broilers challenged with Salmonella enteritidis. Wu et al. [41] demonstrated that supplementing with Bacillus coagulans significantly boosted the BWG and FCR on days 15 to 21 compared to non-supplemented birds. In addition, the changes in microbial populations in the gastrointestinal tract (GIT) caused by probiotics increase the production of short-chain fatty acids (SCFA) and cause immunomodulation, which improves energy metabolism as well [42]. Moreover, it is documented that supplementation of OEO essential oil significantly increased chymotrypsin activity in the digestive system, and improved crude protein digestibility [43] of the active ingredients, which stimulates the digestive system [44] to increase the production of digestive enzymes and also improves the utilization of digestive products through enhancement of the liver functions [45].

Furthermore, the enhancement of FCR due to phytogenic feed additions may be attributed to an increase in the secretions of endogenous digestive enzymes, improving nutrient digestion and gastrointestinal absorption in broilers [46, 47]. Some studies have reported negative or no effects of phytobiotic and probiotic supplementation on gut microflora and growth performance in poultry, which could be due to differences in experimental conditions, such as heat stress [48, 49].

In terms of hematological parameters, both phytobiotic, OEO and the probiotic, *B. subtilis*, dietary treatment groups exhibited a significant increase (*P* < 0.05) in total leukocytic count (TLC) and lymphocyte percentage compared to the control group. The probiotic-supplemented group showed a significantly higher leukocytic count than the phytobiotic group, consistent with previous findings by [50]. Similarly, we demonstrated activation of erythropoiesis with the phytobiotic OEO and the probiotic B. subtilis, as evidenced by increased levels of Hb, RBCs, PCV, and MCH. Our findings align with some previous researches [51, 52], reported an increase in leukocyte value with increasing doses of probiotics from different *Lactobacillus* species and non-*Lactobacillus* species such as *Saccharomyces cerevisiae* [53]. Also, our results were consistent with Khabirov et al. [54] which demonstrated that the probiotic feed additive “Normosil”, containing live cultures of Lactobacillus had a higher number of erythrocytes in the blood at 21 days of age in the experimental groups in comparison to the control. In addition, Hosseini [55] using probiotic, *Saccharomyces cerevisiae*, significantly improved the Hb, RBCs, PCV, Mean Corpuscular Hemoglobin (MCH) and Mean Corpuscular Volume (MCV).

Despite the anatomical separation of the gut and other body systems, a functional interaction between distant sites outside of the gut and intestinal flora has been discovered. Experimental evidence suggests that several microbiota-derived compounds present in the bloodstream contribute to the effect of steady-state hematopoiesis [56]. It has been reported that Hematopoiesis and erythropoiesis were enhanced by oral treatment with lactic acid-producing bacteria (LAB), which also stimulated the release of Stem Cell Factor (SCF) from leptin receptor and bone marrow mesenchymal stromal cells (MSCs) [57]. Several pathways have been proposed to explain the impact of gut microbial metabolites on host homeostasis in tissues beyond the gut. Three mechanisms have been suggested. Firstly, some chemicals produced by microbes may enter the bloodstream and alter immune cell activity in distant target organs. Research by Caballero [58] indicates that pancreatic endocrine cells are modulated by gut microbiota-derived Short-Chain Fatty Acids (SCFAs) to promote the production of essential anti-inflammatory molecules. Secondly, it is possible that microbial compounds in the gut can locally educate immune cells. These educated cells may then migrate to target tissues and influence pathological processes. Previous studies have shown, for instance, that the probiotic Lactobacillus rhamnosus-derived butyrate increases the frequency of intestinal T-reg cells. These cells subsequently migrate to the bone marrow, where they activate osteoblasts through the action of BM CD8 + T cells [59]. Our discovery that lactate derived from the microbiota can influence hematopoiesis in the bone marrow suggests that metabolites from the microbiota may enter the systemic circulation and indirectly affect distant target tissues. Both leukocytic count and hematopoiesis are significantly increased by Origanum essential oil following a similar pattern. These findings are consistent with those of researchers [60] who observed an increase in hematocrit, PCV, hemoglobin (Hb), and the heterophile/lymphocyte (H/L) ratio in blood samples from quail that were administered Origanum essential oil.

The findings regarding intestinal microstructure and morphometry indicate that both probiotic and phytobiotic supplementation had a more beneficial effect on the intestinal health and morphology of the treated groups compared to the control group. The jejunal villus length (VL), width, and crypt depth were significantly increased at 6 and 18 days with the use of probiotic and phytobiotic, and at 28 days with only probiotic supplementation. This improvement can be attributed to the enhanced proliferation of gut epithelial cells and the activation of mitotic cell division, both of which contribute to improved intestinal function and growth enhancement effects produced by the phytobiotic OEO and the probiotic *B. subtilis*. Our results agreed with several researchers using *B. subtilis* probiotic [49], Abd El-Moneim and Sabic [61–63] or phytobiotic, OEO Banerjee et al. [64, 65] who also found effective improvement in intestinal morphology following supplementation with B. subtilis probiotic or phytobiotic OEO. This improvement can be attributed to the ability of probiotics and phytobiotics to inhibit intestinal colonization by pathogenic bacteria, enhance epithelial barrier integrity, produce antimicrobial substances like bacteriocins, reduce the production of toxic compounds that damage intestinal epithelium, inhibit villus destruction, and stimulate epithelial cell proliferation [62], Hashemi et al. [65–67]. Moreover, Yu et al. [68] demonstrated that probiotic bacteria such as Lactobacillus and Bifidobacteria stimulate the production of beneficial bacterial metabolites, which increase the villus height to crypt depth (VH: CD) ratio.

Additionally, the present study provides insight into the humoral immune response of chicken supplemented with probiotic and phytobiotic feed additives by detecting antibody titers against the Newcastle disease virus in serum using hemagglutination inhibition test. The result indicated that antibody titers against ND were significantly higher (*P* < 0.05) in phytobiotic-fed groups on both days 18 and 28 compared to probiotic and control groups. This beneficial effect of phytobiotic on the immune response may be attributed to the significant increase in the bursa of Fabricius weight index in chickens that received phytobiotic compared to the control group [69]. The increase in immune tissue weight produces an effect on immune cell phenotypes, immune cell proliferation, and antibody production [70] explaining the significant improvement in antibody titers against NDV in chickens supplemented with phytobiotic. There is a strong correlation between the relative size of the bursa and the average levels of antibody expression [71]. Furthermore, Awaad et al. [72] revealed that the immune-stimulatory effect of essential oils, a main component of phytobiotic, may stimulate complement receptor-mediated phagocytosis, leading to a significant increase in humoral antibody titers against NDV. Studies have shown that B. subtilis can increase systemic IgG and mucosal IgA titers in chickens immunized with live-attenuated or inactivated forms of pathogens such as NDV, AIV, and Salmonella [73, 74] Also, broilers fed OEO at 300 ppm produced higher secondary total antibody titers against sheep red blood cells (*P* < 0.01) and their Immunoglobulin G titer was higher (*P* < 0.05) than those fed the control diet. These results similar to findings obtained by Hosseini et al. [75] revealed that the phytobiotic failed to create a significant improvement in the humoral immunity of broiler chicks. Also, the findings of Talebi et al. [76] supported our results since they found that feeding probiotics improved antibody titers against viral diseases like Newcastle disease (ND) and Infectious bursal disease (IBD).

The study demonstrated that both phytobiotics and probiotics led to a significant increase in phagocytic percentage and index compared to the control group. These findings are consistent with [77] who observed enhanced immune responses in broiler chickens with increased serum NDV antibody titers, phagocyte percentage, and phagocytic index following probiotic supplementation. Similarly, Yu et al. [68] highlighted the immune-boosting effects of probiotic bacteria such as Lactobacillus and Bifidobacteria, which enhanced phagocytosis, pro-inflammatory cytokine production, intraepithelial lymphocyte numbers, and antigen-specific antibody development in broiler chickens. also reported improved lymphocyte proliferation, phagocytosis, serum antibody levels, and complement components against viral infections in broiler chickens supplemented with essential oils.

Furthermore, the study revealed a significant increase (*P* < 0.05) in lymphocyte proliferation on the 18th and 28th days with phytobiotic administration compared to the control group. Our findings were similar to that of Revajova et al. [78] who observed increased lymphocyte proliferation following supplementation with oregano and sage extracts in chickens. Also, Li et al. [79] found that essential oil supplementation improved serum lymphocyte proliferation rate, phagocytosis rate, immunoglobulin (Ig) G, IgA, IgM, C3 and C4 levels in piglets. Also, in aquaculture. Aly et al. [80] recorded significant improvement in hemoglobin (HB), red blood cells (RBCs) and white blood cells (WBCs) count, lysozyme, phagocytic activity, nitric oxide levels and cytokines up-regulation in *Nile Tilapia*, supplemented with 2% oregano oil. Moreover, Phytogenic compounds also induce their immunomodulatory effects by increasing immune cell proliferation, rising cytokines expression and elevation of antibody titers [81–83]. This improvement is most likely due to the presence of thymol and carvacrol in the essential oil [84]. The organosulfur components of essential oil have been demonstrated to promote macrophage phagocytosis and to stimulate macrophage chemotaxis, human neutrophil responses with ROS generation, and lymphocyte proliferation [85, 86]. Moreover, carvacrol and thymol, the constituents in OCE showed mild to moderate inhibition of phagocytosis (25–40% inhibition at doses ranging from 40 µg/mL to 60 µg/mL) while the highest inhibitory activity was found (72% at 56 µg/mL) [87]. On the other hand, Du et al. [88] did not find any remarkable effect of essential oils (EO, which contained 25% thymol and 25% carvacrol as active components) supplementation on the proliferative responses of T cells and B cells *in-vitro.*

## Conclusion

The current study revealed that, the growth enhancing effects following the supplementation with *B. subtilis* & oregano essential oil seems to closely relate to a healthy intestinal barrier expressed by increased VH, and VH/crypt depth (CD) ratio, which provides better chances of nutrient absorption and is an indicative parameter for better gut morphology promoted epithelial cell proliferation. Also, immunostimulant effects following the same supplementation seems attributed to humoral & innate immunity activation.

## Data Availability

We confirm that all the data supporting the finding are available in the article.
